# Photo-Induced Cross-Linking of Unmodified α-Synuclein Oligomers

**DOI:** 10.1021/acschemneuro.3c00326

**Published:** 2023-08-25

**Authors:** Lei Ortigosa-Pascual, Thom Leiding, Sara Linse, Tinna Pálmadóttir

**Affiliations:** Department of Biochemistry and Structural Biology, Lund University, 221 00 Lund, Sweden

**Keywords:** Parkinson’s disease, cross-linking, self-assembly, structure, organization

## Abstract

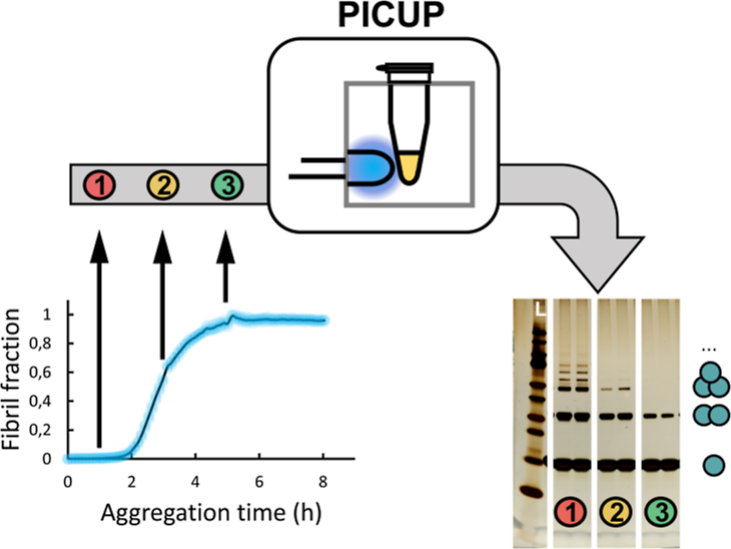

Photo-induced cross-linking of unmodified proteins (PICUP) has been used in the past to study size distributions of protein assemblies. PICUP may, for example, overcome the significant experimental challenges related to the transient nature, heterogeneity, and low concentration of amyloid protein oligomers relative to monomeric and fibrillar species. In the current study, a reaction chamber was designed, produced, and used for PICUP reaction optimization in terms of reaction conditions and lighting time from ms to s. These efforts make the method more reproducible and accessible and enable the use of shorter reaction times compared to previous studies. We applied the optimized method to an α-synuclein aggregation time course to monitor the relative concentration and size distribution of oligomers over time. The data are compared to the time evolution of the fibril mass concentration, as monitored by thioflavin T fluorescence. At all time points, the smaller the oligomer, the higher its concentration observed after PICUP. Moreover, the total oligomer concentration is highest at short aggregation times, and the decline over time follows the disappearance of monomers. We can therefore conclude that these oligomers form from monomers.

## 1 Introduction

The formation and accumulation of amyloidogenic proteins into stable aggregates of low solubility, called amyloid fibrils, are connected to many diseases such as: type II diabetes,^1^ Parkinson’s,^2^ Alzheimer’s,^3^ and prion diseases.^4^ The formation of the amyloid fibrils involves the generation of smaller transient species called oligomers. The cytotoxicity of the oligomeric species^5−7^ makes them an important target to study. However, their transient nature, heterogenicity, and low concentration relative to monomeric and fibrillar species impose significant experimental challenges.^8^ Cross-linking of oligomers has been found to be useful for overcoming those challenges.

Cross-linking methods promote the formation of covalent bonds between or within molecules. Since the introduction of the cross-linking technology in 1958,^9^ countless innovative cross-linking methods have been developed. Chemical cross-linking is most commonly performed with the use of so called cross-linkers, which are molecules with various functional groups, which react with the target molecules to form covalent bonds.^10^ The most frequently used ones include amine-reactive groups and sulfhydryl-groups. For instance, in protein studies, cross-linkers that react with primary amines such as lysine side chains and amino termini are commonly used.^11,12^ Alternatively, light can be used to perform photo-induced cross-linking by triggering the activation of an otherwise inert reagent,^13^ such as photo-reactive artificial amino acids^14^ and metal-activated redox reactions. The most prominent example of the latter, and the method of interest in this paper, is the photo-induced cross-linking of unmodified proteins (PICUP), an oxidative coupling method initially invented to study proteins that form stable oligomers.^15^

PICUP was first demonstrated in 1999 in the study of the hexameric recombination protein uvsY,^15^ followed by many studies using the technique to study the oligomeric pattern of various other proteins.^16−22^ Since then, PICUP has been used for diverse purposes, including the mapping of protein–ligand interactions,^23−26^ the labeling and topographic study of G-coupled receptors,^27^ the preparation of covalently bound dimeric monoclonal antibodies,^28^ and for the polymerization of poly(l-tyrosine) silica particles.^29^ Furthermore, this method has been found to be especially useful for studying oligomerization of amyloidogenic proteins, such as insulin,^30,31^ prions,^32−35^ human transthyretin,^36^ Tau,^37^ α-synuclein,^38−46^ and amyloid β (Aβ).^34,42,47−78^

The reagent that makes PICUP photo-sensitive is ruthenium (II) tris-bipyridilcation [Ru(bpy)], a metal-coordinated complex. Ru(bpy) has an absorption maximum in aqueous solution at 452 nm. When it absorbs light and gets into an excited state, it donates an electron to an electron acceptor such as ammonium persulfate (APS). The newly formed oxidant Ru(III)bpy_3_^3+^ extracts an electron from a nearby protein, forming a highly reactive protein radical. This protein radical can then subsequently react with other protein molecules. The reaction can be stopped by addition of a reducing agent such as β-mercaptoethanol or dithiothreitol. The reaction is mainly conditioned by the protein structure, the distance between reacting residues, and the capacity of the groups to stabilize an unpaired electron.^15,47,53^ Early studies used a lamp as the light source, with the sample inside a camera house, using the shutter to control the lighting time down to 1 s.^47,53^ Later improvements have incorporated manually controlled light-emitting diode (LED) with the sample tube placed in a box for more precise wavelength and sample illumination.^79−81^

Photo-cross-linking methods tend to have a greater specificity when compared to chemical cross-linking due to the short lifetime of the photo-induced intermediate radicals.^14^ Another factor favoring specificity is the reaction time, which is substantially shorter for PICUP than other cross-linking methods, with PICUP having been shown to be effective with irradiation time of less than a second.^15,20,23,38,47,51−53^ While photo-cross-linking is mostly carried out through the addition of a photoreactive group in the target molecule,^82,83^ PICUP makes use of an external metal-coordinated complex to induce a redox reaction. The procedure needs no modifications of the protein of interest, ensuring the system being the closest possible to the native state prior to the reaction.^56^ The light used to trigger the reaction is in the visible range, hence, non-damaging to cells or other macromolecules. This makes the method applicable to cell extracts.^20^ Finally, the outcome of the cross-linking is a single covalent bond between two macromolecules. The fact that residues must be very close in space to react together makes the method a “zero-length” cross-linker and ideal for determining contact sites as molecules must be at covalent bond distance from each other to form a cross-link.^84^

PICUP is a useful method for the characterization of the size and distribution of the transient oligomers that form during amyloid fibril formation.^15,53^ PICUP can “trap” transient oligomeric species by cross-linking monomers within the same oligomer. Additionally, PICUP allows the analysis of the oligomers in their native state, without any attached cross-linker that could alter the oligomeric structures or affect the amyloid fibril formation process. PICUP has therefore been used for comparing oligomer distribution of native and mutant species,^30,32,38,39,47−49,55,66,71^ evaluating the effect of inhibitors or other factors on oligomer formation,^31,34,40,42−44,52,54,58,60−63,65,67,72,73,77^ generating covalent oligomers of defined size,^33,35,64,69,70,75^ developing methods to detect covalent dimers in patients,^74^ understanding metal binding to α-synuclein,^45,46^ and comparing different Aβ sources.^76,78^

**Figure 1:**
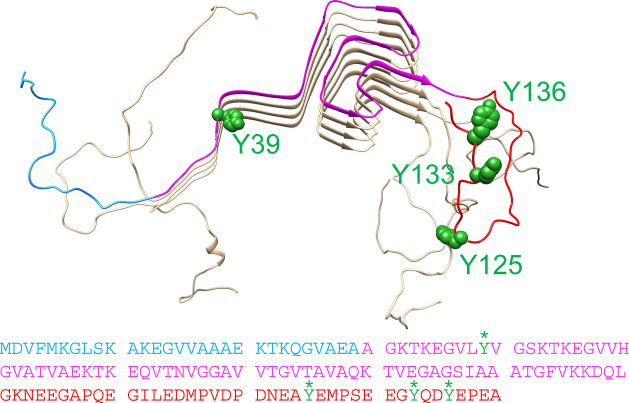
Top: solid-state NMR model of the structure of full-length human α-synuclein fibril. Bottom: α-synuclein sequence. The colors are used to highlight the N-terminal tail (blue), fibril core (pink), and C-terminal tail (red) of one full-length α-synuclein molecule, aligned with the fibril core of other α-synuclein molecules (tan). The four tyrosine residues Y39, Y125, Y133, and Y136 [green; with displayed side chain in the structure, marked with a star (*) in the sequence] are shown because Y is the residue type most prone to react via PICUP. This figure was prepared using Chimera^109^ based on the pdb file 2N0A.^90^

α-Synuclein is a 140 residue long and intrinsically disordered protein with a molecular weight of 14.5 kDa.^85^ The formation of α-synuclein amyloid fibrils and their accumulation into inclusion bodies, named Lewy bodies, has been found to be the hallmark of Parkinson’s disease.^86^ The protein is divided into three regions,^87^ the N-terminal region (amphipathic), the central hydrophobic region (also named the NAC region), and the C-terminal region (acidic).^87−89^ Residues ∼29 to 100 form the core of the fibril structure.^90−92^ The protein sequence contains four tyrosine residues (Y), one in the N-terminal region (Y39) and three in the C-terminal region (Y125, Y133, and Y136) (Figure 1). This makes α-synuclein an ideal target for PICUP analyses as tyrosine has been found to be one the most reactive residues for the cross-linking reaction besides tryptophan.^30,41,47,53^ Additionally, dityrosine cross-linked α-synuclein has been found in Lewy bodies in post-mortem tissue from Parkinson’s disease patients,^93^ indicating that PICUP products are biologically relevant. The aggregation of α-synuclein is highly dependent on pH.^94^ While the aggregation at physiological pH is overall slower and dominated by elongation, at mildly acidic pH (below pH 6), the reaction has been found to be dominated by surface-catalyzed secondary nucleation, with the rate of secondary nucleation being about 10^4^ times faster than that at physiological pH.^94,95^ Formation of oligomers has been found to be related to secondary nucleation;^96,97^ therefore, the experiments were performed at pH 5.5, where the secondary nucleation of α-synuclein is prominent and oligomerization is most likely maximized. To the best of our knowledge, the cross-linking of α-synuclein by PICUP below pH 6 has not been studied before.

The current study concerns the application of PICUP to the analysis of oligomer distributions at different time points during an amyloid formation process or under different conditions. We aim to expand the knowledge, usefulness, and understanding of PICUP for amyloid protein research, using α-synuclein as a model system. Toward these aims, we have designed a reaction chamber and optimized the reaction conditions, making the method more reproducible and accessible, as well as enabling the use of shorter reaction times compared to previous studies. The results highlight the strengths and limitations of the method. Furthermore, oligomeric populations formed during aggregation of α-synuclein at pH 5.5 were studied at the optimized conditions using the newly designed reaction chamber.

## 2 Results

### 2.1 Evaluating PICUP of α-Synuclein

#### 2.1.1 Building the PICUP Reaction Chamber

**Figure 2:**
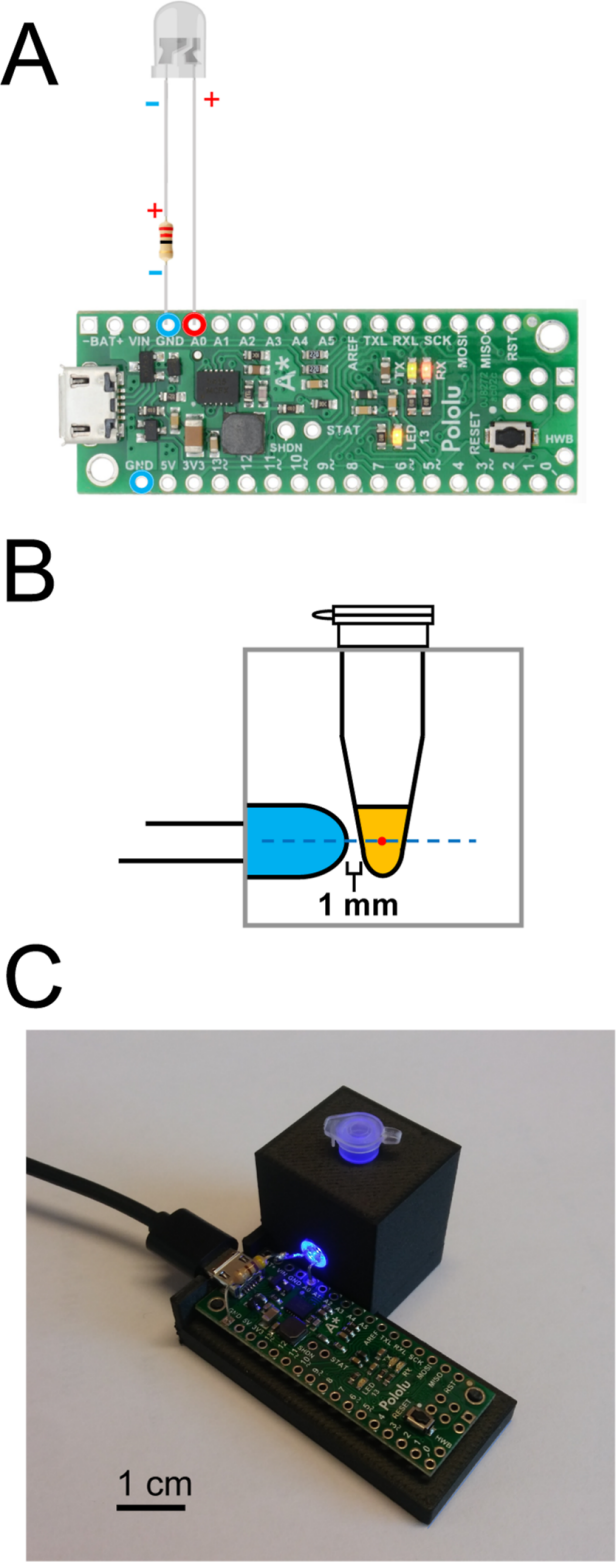
Reaction chamber designed for PICUP. (A) Schematic representation of PololuTM A-Star 32U4 Mini SV (ac02c) board with the resistor in contact with the ground (blue circle) and the positive leg of the LED in contact with the A2 port (red circle). (B) Drawing of the side view of the reaction chamber, with the center of the LED aligned with the center of a 20 μL sample, 1 mm away from the sample tube. (C) Photograph of the mounted reaction chamber.

Good control of the reaction conditions is crucial for high reproducibility and optimization of the method. To this end, we designed and built a reaction chamber that would allow for control of the light exposure time of the sample with millisecond accuracy (Figure 2). The reaction chamber was designed to fit a PCR tube, in which a 20 μL sample could be placed. Perpendicular to that, an LED is inserted, located 1 mm from the tube wall. This minimizes potential intensity loss due to the distance between the light source and the sample. Once the reaction chamber was designed and 3D-printed, the electronics and the LED were fitted to it. An Arduino program was made to control the lighting time with millisecond accuracy.

The PICUP reaction of α-synuclein using the designed reaction chamber was evaluated by performing the experiments in the presence and absence of APS, Ru(bpy), and light (Figure S1). This verified that the reaction only proceeds when both APS and Ru(bpy) are present, and sufficient light is illuminating the sample. We find that the outcome of the PICUP varies with lighting time, in accordance with previous studies (see Section 2.1.2).^15,38,47^

#### 2.1.2 Effect of Lighting Time in PICUP of α-Synuclein

**Figure 3:**
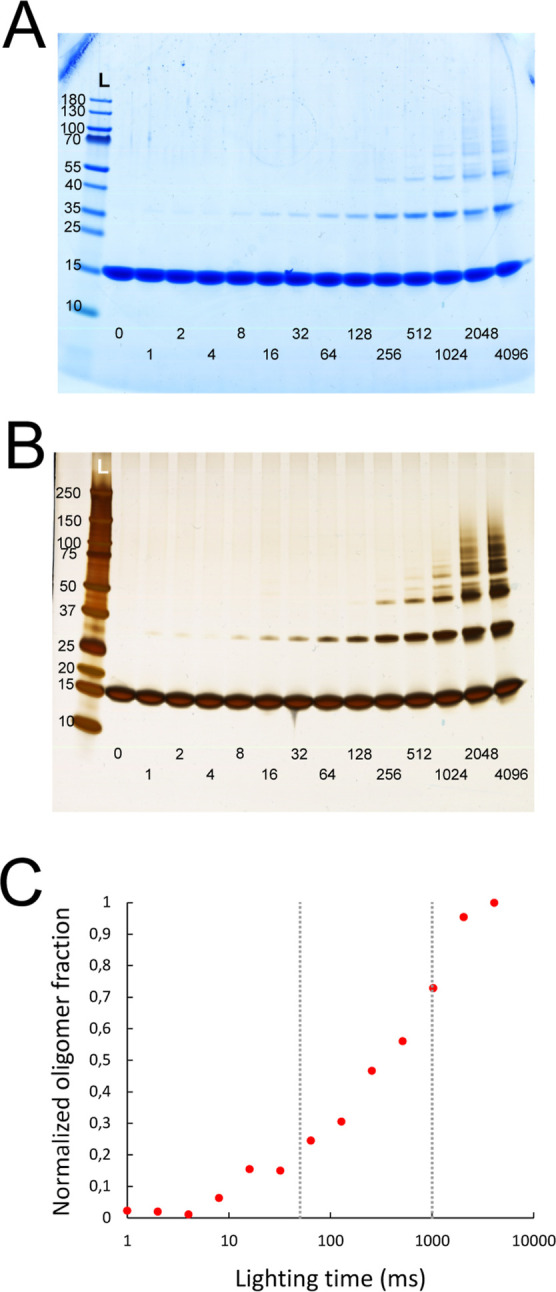
Photo-induced cross-linking of α-synuclein under different lighting times. Freshly purified α-synuclein was cross-linked at different lighting times and run in two gels, stained with either InstantBlue (A) or silver-staining (B). The number under each lane indicates the lighting time in ms. Gel band intensity of the silver-stained gel was measured with ImageJ, and the fraction corresponding to oligomers was calculated for each lighting time. These values were normalized relative to the highest oligomer fraction and plotted over lighting time in a logarithmic scale (C). The dotted gray lines indicate the two lighting times chosen for later studies, 50 ms and 1 s.

In order to better understand the effect of the lighting time on PICUP of α-synuclein, we performed the reaction with a range of lighting times between 1 and 4096 ms (Figure 3). Without any irradiation, there was no sign of species other than monomeric α-synuclein (∼15 kDa) being present, even with the intensity of the image increased to the maximum (Figure S2). Interestingly, 1 ms lighting time was enough to generate a visible band at a position corresponding to the size of a dimer (∼30 kDa). This shows that PICUP of α-synuclein is a quite efficient reaction sensitive to a small fraction of the dimer. Further increasing the lighting time leads to the appearance of more oligomeric species, albeit the bands become more blurred. This is likely due to additional covalent bonds being formed during longer light exposure, leading to a more varied morphology of the cross-linked oligomer population and thus different mobilities of oligomers of the same association number.

One would expect that as more oligomers are cross-linked, less α-synuclein should be left to migrate as a monomer on the gel. However, we note that the monomer band intensity does not seem to visibly change regardless of the lighting time. Monomers within fibrils dissociate in the SDS-loading buffer and therefore end up as monomers on the gel. As oligomers are a small fraction of the sample in solution, cross-linking all of them would still leave most of the sample to migrate as monomers, representing the sum of monomers in solution and in fibrils. This means that the difference in the monomer band intensity between no cross-linking and cross-linking is not substantial. On top of that, gels were slightly overloaded to observe the less populated oligomer bands. This caused the monomer band to be saturated, and thus, any changes in its population being even less correctly represented.

The intensities of the gel bands were measured with ImageJ (Figure S3). The oligomer intensity at each lighting time was normalized for the loading of the gel by dividing the intensity of bands of oligomer sizes (∼30 kDa band and above) by the intensity of all bands found on the same lane. To compare the effect of different lighting times, we related the oligomer fraction at each lighting time to the highest one (at lighting time = 4096 ms) (Figure 3C).

It is crucial to note that the α-synuclein employed for these measurements was purified via size exclusion chromatography, and all samples were kept for approximately 0.5–1 h on ice until the PICUP reaction was performed (see methods Section 4.3.). This procedure is often used to ensure that the protein is in fully monomeric state prior to other experiments. However, we observe that PICUP of this sample leads to the formation of a low population of cross-linked oligomeric species, visible on both the silver-stained and InstantBlue-stained gel (Figure 3A,B).

#### 2.1.3 Is Freely Diffusing α-Synuclein Being Cross-Linked?

**Figure 4:**
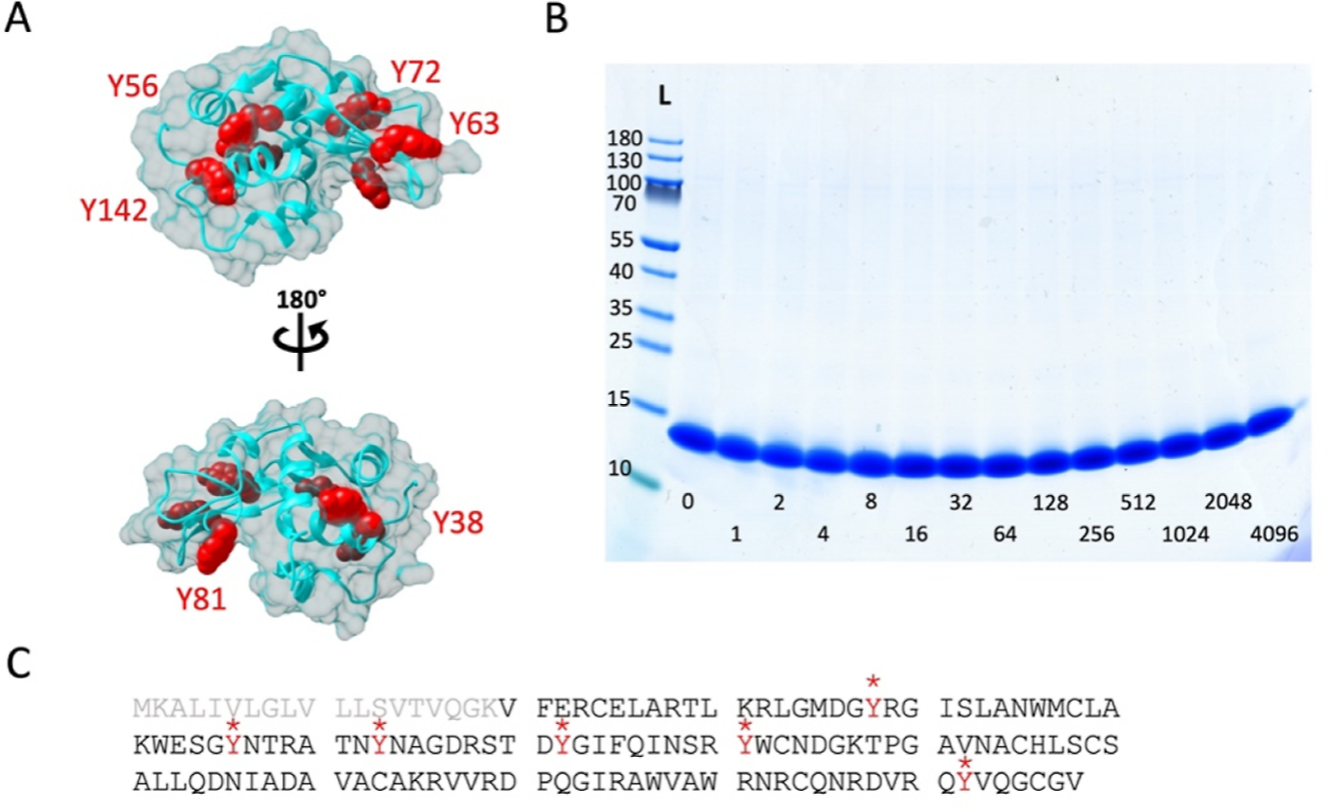
Control for potential cross-linking of diffusing species using human lysozyme. (A) X-ray structure of residues 19–148 of human lysozyme. The six tyrosine residues, in the protein surface, are colored red. This figure was prepared using Chimera^109^ based on the pdb file 1LZ1.^110^ (B) Photo-induced cross-linking of lysozyme under different lighting times. The number under each lane indicates the lighting time in ms. (C) Human lysozyme sequence with the tyrosine residues Y38, Y56, Y63, Y72, Y81, and Y142 highlighted in red. The parts of the sequence in gray are the residues missing in the structure shown in panel (A).

To evaluate whether the PICUP products we observe after incubation of monomers on ice come from transient oligomers or diffusing species, we used as a control a non-oligomeric protein of a similar size to α-synuclein, human lysozyme, with 148 amino-acid residues, six of which are tyrosines (Figure 4). The six tyrosines are located at the surface of the protein, accessible for potential cross-linking. However, when performing PICUP under the same conditions as for α-synuclein, only very faint and constant bands for species above monomer size were detected for lysozyme (Figures 4B and S4). With lysozyme having a similar molecular weight and with more reactive residues than α-synuclein, the lack of cross-linking of lysozyme supports the idea that the species observed during PICUP of α-synuclein are indeed oligomers and not monomers being cross-linked due to diffusion into proximity of one another.

#### 2.1.4 Evaluation of the Termination of the Reaction

**Figure 5:**
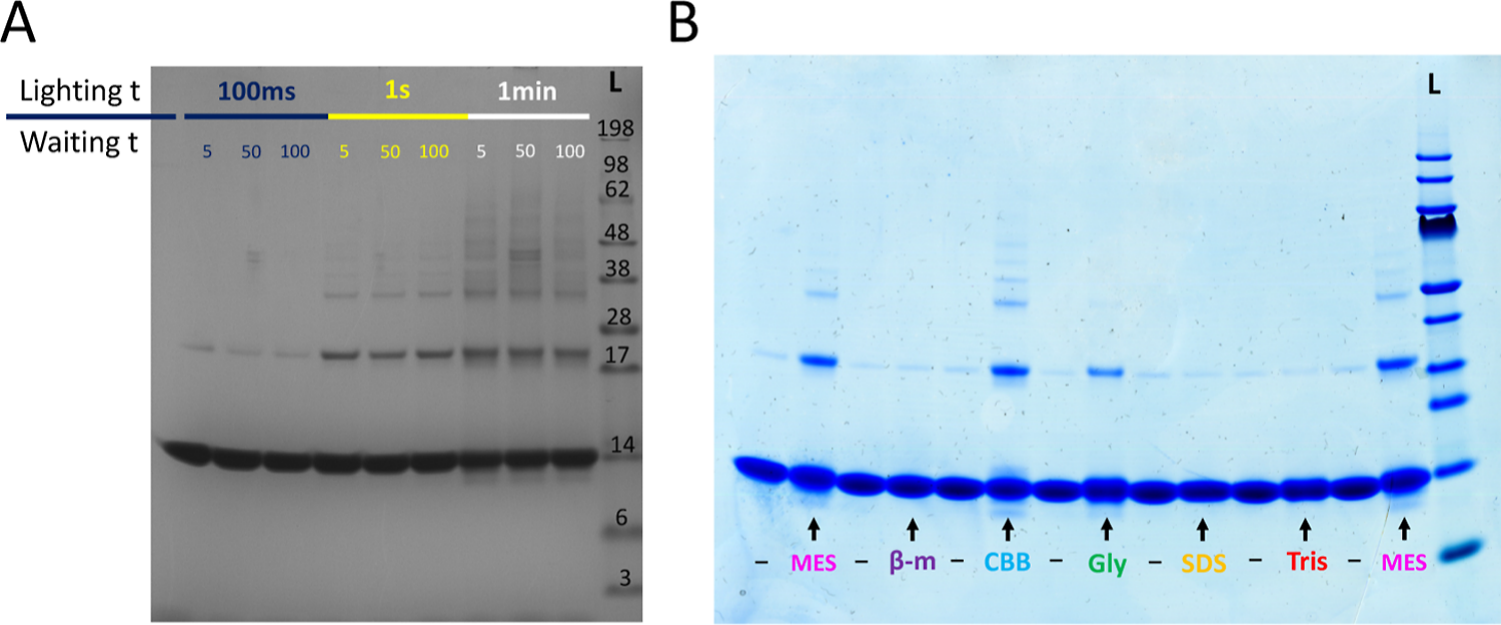
Termination of the reaction and the effect of the 5× STOP solution ingredients. (A) Effect of quenching time in cross-linking of α-synuclein. The reaction was performed with lighting times of either 100 ms, 1 s, or 1 min, as indicated on the top of the gel. Once the lighting time was over, the 5× STOP buffer was added to the solution after a waiting period of either 5, 50, or 100 s. (B) Effect of the 5× STOP buffer ingredients. For the lanes labeled “–”, PICUP was performed with 50 ms lighting time and then stopped. For the lanes labeled “MES”, the reaction was carried out with 50 ms lighting time, and after addition of 10 mM MES/NaOH, 0.02% (w/v) NaN_3_, pH 5.5, the reaction was triggered again for an additional 10 s. To evaluate the effect of each 5× STOP reagent in stopping α-synuclein from further reacting, the same procedure for “MES” was followed but by adding the individual reagent instead of the MES buffer. This way, β-mercaptoethanol (β-m), coomassie brilliant blue (CBB), glycerol (Gly), and sodium dodecyl sulfate (SDS) or 4.5 M Tris pH 8.45 (Tris) were added to reach a final concentration equal to the one found in the 5× STOP buffer for the same ingredient, and then, the sample was reacted for additional 10 s.

After observing the high sensitivity of PICUP toward lighting time, we set out to investigate the sensitivity of the method to the termination of the reaction (Figure 5). In the previously performed experiments, the 5× STOP buffer (see Section 4.7.1) was added immediately (within ∼3 s) after the light was turned off. To test whether the time passed between the lighting and the addition of the 5× STOP buffer had any effect on the observed PICUP product, we compared waiting for 5, 50, or 100 s before stopping the reaction. Regardless of the lighting time used for the reaction (100 ms, 1 s, or 1 min), we found no difference in the species distribution by SDS PAGE over the different reaction stopping times. This implies that the concentration of reactive species decreases fast enough that adding the stopping buffer after 5 or 100 s does not make any difference. Therefore, the reaction ends before addition of the 5× STOP buffer, and adding it merely prevents any further reaction upon exposure to light during continued sample handling.

We then tested the ability of each ingredient of the 5× STOP buffer in preventing the reaction from further happening. To do so, we performed the reaction on the protein using 50 ms lighting time, added a solution, and then applied light for 10 s more. When the added solution was 2-(*N*-morpholino)ethanesulfonic acid (MES) buffer, the additional 10 s reaction led to the appearance of more bands. However, when β-mercaptoethanol was added, the additional 10 s reaction did not produce more bands. This was to be expected as β-mercaptoethanol is the reducing agent in the 5× STOP buffer, which serves to stop the reaction by reducing the Ru(bpy) oxidant (Ru(III)bpy_3_^3+^). Interestingly, other reagents, such as SDS or Tris, seem to prevent further reaction from happening, potentially by altering the oligomeric state of the protein or affecting the reagents themselves. This means that the reaction stopping solution may not need to contain a reducing agent such as β-mercaptoethanol.

### 2.2 PICUP of α-Synuclein under Aggregating Conditions

#### 2.2.1 Effect of ThT in PICUP of α-Synuclein

Once the PICUP of α-synuclein was optimized, we decided to apply it under conditions where secondary nucleation is favored [20 μM α-synuclein in 10 mM MES/NaOH, 0.02% (w/v) NaN_3_, pH = 5.5] using two lighting times in parallel (50 ms and 1 s).

**Figure 6:**
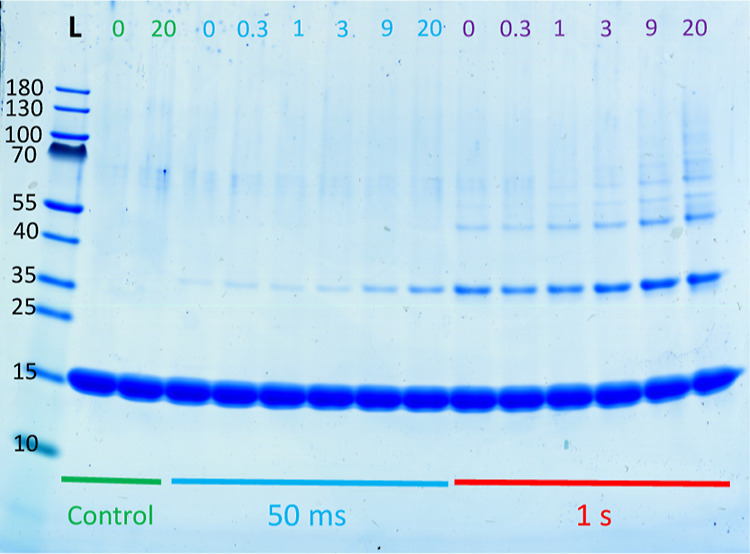
Effect of ThT on the PICUP of α-synuclein. α-Synuclein was cross-linked in the presence of different concentrations of ThT (0, 0.3, 1, 3, 9, and 20 μM), indicated at the top of each lane. The reaction was performed with both 50 ms (blue line) and 1 s (red line) lighting times. Two controls with either 0 or 20 μM ThT were performed where buffer was added instead of the PICUP reagents, and no light was applied (green line).

First, the effect of the presence of ThT on the PICUP product was tested (Figure 6). Being the most common fluorescence dye used to follow amyloid protein aggregation, its effect on the oligomer population is critical to know. We observed that the intensity of the oligomer bands increased with ThT concentration for both lighting times, especially at 9 and 20 μM ThT. This implies that the presence of ThT leads to the cross-linking of more oligomers. This could be either a direct effect of ThT on the oligomer population or an indirect effect of ThT on the PICUP reaction. The presence of ThT did not induce formation of extra bands for lysozyme (Figure S5). Based on these results, we decided to continue our studies with 3 μM ThT to minimize its effect on the observed oligomer patterns after the PICUP reaction.

#### 2.2.2 α-Synuclein Aggregation as a Function of Time

**Figure 7:**
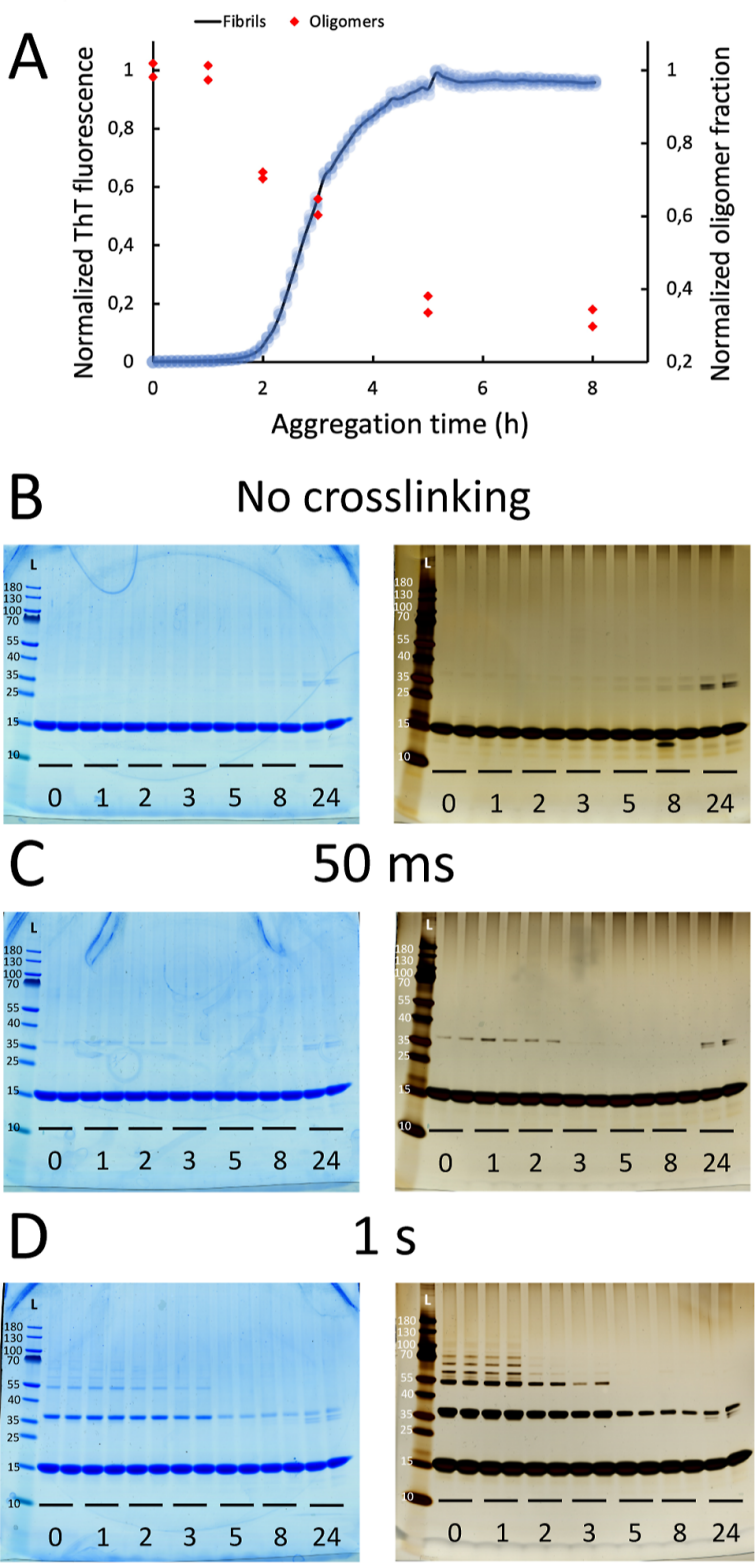
PICUP of α-synuclein at different points throughout its aggregation. α-Synuclein was aggregated in the presence of seeds at 37 °C under quiescent conditions and monitored by ThT fluorescence (A). The ThT fluorescence of six samples was normalized (light blue dots), and their average was plotted as a function of time (black line). Oligomer fractions (red dots) are normalized relative to the average value at *t* = 0. Samples were collected at *t* = 0, 1, 2, 3, 5, 8, and 24 h. Six samples were collected at each of these points and divided in three sets of duplicates. One duplicate set was left un-cross-linked (B), one was cross-linked for 50 ms (C), and one cross-linked for 1 s (D). Each sample was analyzed in two SDS-PAGE gels and stained with either InstantBlue (left) or silver staining (right). The 1 s PICUP silver-stained gel was used for ImageJ analysis of the bands. The oligomer fraction was calculated by dividing the intensity of oligomer bands by the total intensity of all the bands in the same lane, to normalize for the loading of the gel. Finally, to look at the time evolution of the oligomer fraction, these values were normalized relative to the average of the ones at *t* = 0 h and plotted together with the normalized aggregation curve (A).

The aggregation of α-synuclein starting from 20 μM monomer supplemented with 200 nM fibril seeds at 37 °C was monitored by following the fluorescence (Figure 7A) of 3 μM ThT. Samples were taken at different time points during the aggregation process: at the start (*t* = 0 h); the middle of the lag phase (*t* = 1 h); the transition from the lag phase to the exponential phase (*t* = 2 h); the half time (*t* = 3 h); the transition between the exponential phase and the final plateau (*t* = 4 h); the final plateau (*t* = 8 h); and an additional point further into the plateau (*t* = 24 h).

#### 2.2.3 Analysis of the α-Synuclein Aggregation Process without Cross-Linking

**Figure 8:**
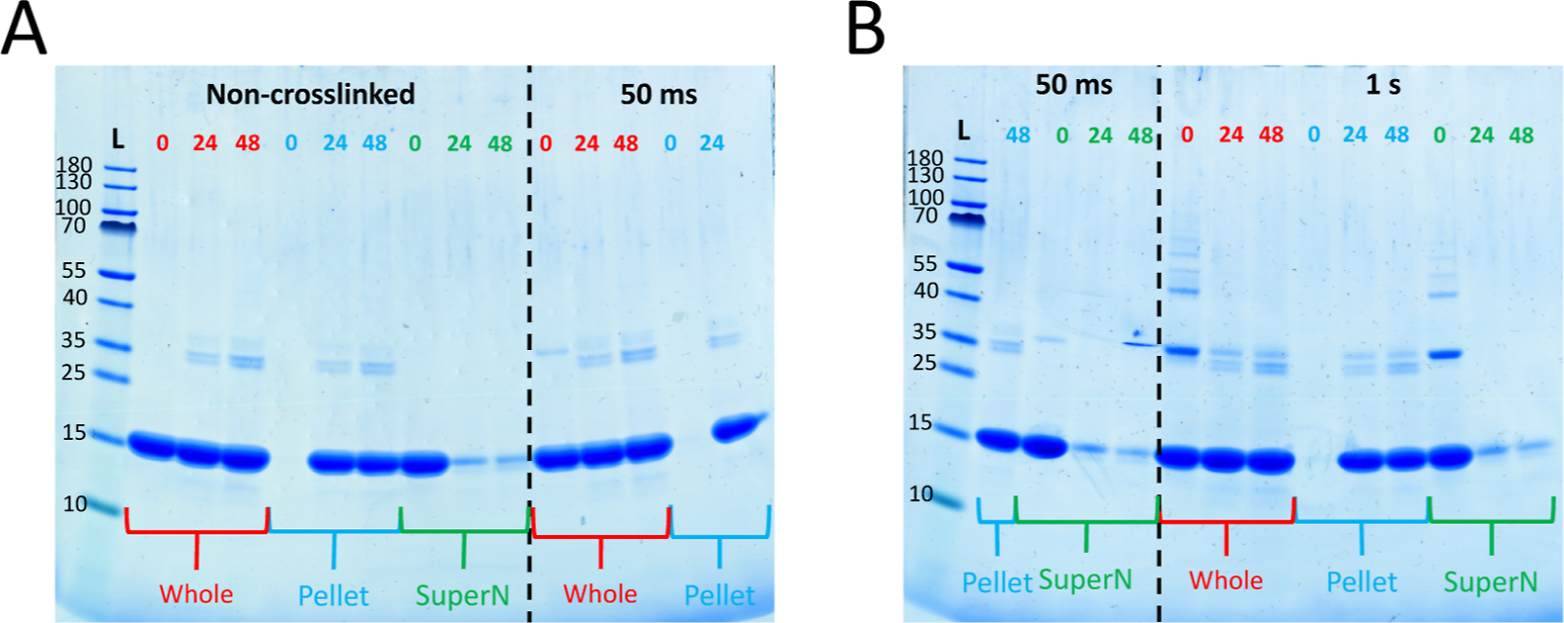
Separation of α-synuclein samples via centrifugation. 100 μL of samples was collected before, after 24, and after 48 h of aggregation (labeled 0, 24, and 48, respectively). Some of these samples were centrifuged in order to separate the fibrils from the soluble species. 80 μL of the supernatant was collected (SuperN, green), and the remaining supernatant was discarded. The pellet was resuspended in 100 μL of MES buffer and centrifuged again. After getting rid of the supernatant, the pellet was resuspended in 100 μL of MES buffer again (pellet, blue). Some of the samples were left uncentrifuged (whole, red). Samples were then either cross-linked for 50 ms, for 1 s, or not cross-linked at all (50 ms, 1 s, and non-cross-linked, respectively). After adding 5× STOP buffer to all of them, the samples were analyzed with SDS-PAGE.

Some of these samples from the aggregation time course were analyzed by SDS PAGE prior PICUP (Figure 7B). In these cases, gel bands appeared only at the position corresponding to the monomer size (∼15 kDa) and did not vary throughout the aggregation, except at the very end. Due to the denaturing nature of the sodium dodecyl sulfate–polyacrylamide gel electrophoresis (SDS-PAGE), any potential fibrils are dissolved into monomers, supported by the fact that no new species with the size of the fibril can be seen in the wells at the top of the gel. This means that the band we observe at the monomer size corresponds at all times to the sum of monomers and fibrils. This was corroborated by separating fibrils and species in the solution phase via centrifugation (Figure 8A, “non-cross-linked”), after which the monomer-sized band (∼15 kDa) appears in the solution fraction at the beginning of the aggregation but in the fibril fraction after aggregation (24 and 48 h).

A very faint band of approximately dimer size (above the 35 kDa marker) can be seen throughout all samples. This band represents a very minor population of the sample and stays constant throughout the aggregation time course.

Two bands migrating as approximately 30 kDa in size (below the 35 kDa marker) appear toward the end of the aggregation and become clearly visible at the plateau (*t* = 24 h). We note that these extra bands are only detected after fibril formation and with slightly higher migration (lower apparent *M*_w_) than the cross-linked dimer band. These bands seem to follow the fibril fraction upon centrifugation (Figure 8). MS analysis verified that the protein found in these bands is indeed α-synuclein but with a truncation at Asp-119 (MS data can be found in Supporting Information). Other investigators have detected this truncation and consider it a common product of the metabolism of α-synuclein, which can be found in both normal and disease brains with mass spectrometry.^80,98,99^

#### 2.2.4 Analysis of the Aggregation Process with PICUP

The samples from the aggregation time course were also analyzed by SDS PAGE after PICUP (Figure 7C,D). Samples cross-linked for 50 ms and 1 s show a clear presence of oligomers at *t* = 0 h, in agreement with the data above (Figure 3). While with 50 ms lighting time only the dimer band can be seen, more oligomers show up with 1 s lighting time. The oligomers cross-linked with PICUP can be seen to gradually decrease in concentration as the aggregation reaction proceeds. With 1 s lighting time, a dimer band is observed all the way until after 24 h, albeit fainter than that at the beginning. This could come from either the small population of oligomers at equilibrium with the fibril, or from α-synuclein being cross-linked within the fibril. Interestingly, the highest population of oligomeric species seems to be present during the lag phase (*t* = 0 h and *t* = 1 h) and starts decreasing as the exponential phase begins to be visible (*t* = 2 h). With 1 s lighting time, a continued decrease in oligomeric species can be observed after the half time (*t* = 3 h). These analyses were performed for the entire reaction mixture. In other experiments, cross-linked samples were also centrifugated between PICUP and SDS PAGE (Figure 8, “50 ms” and “1 s”). As above, fibrils are seen to dissolve in the SDS PAGE loading buffer, showing that the observed monomer band originates from both monomers and fibrils. At time point zero, the oligomers are present in the supernatant. However, after 24 and 48 h, the oligomers (dimers) are present in the pellet fraction.

The gel bands obtained with 1 s lighting time and stained with silver staining (Figure 7D, right) were used for ImageJ analysis. The oligomer fraction was calculated by dividing the intensity of the oligomeric bands by the total band intensity of the same gel lane. To visualize the time evolution of oligomers, we relate the values from all time points to those obtained at time 0 (Figure 7A).

## 3 Discussion

The reaction chamber and the optimization of the procedure has allowed us to prove that precise control of the lighting time is a crucial factor when using PICUP. Due to the possible bias of lighting times, we find the parallel use of a low and high lighting time ideal for comparative studies. With these conditions, we have been able to observe the behavior of cross-linked α-synuclein oligomers throughout its aggregation process. Our results show that these oligomers are in a fast equilibrium with monomers, and their disappearance follows the appearance of fibrils.

### 3.1 Improvement of the Method and the Reaction Chamber

PICUP is potentially a quite powerful method, but its usefulness has been lowered by the difficulty of obtaining reproducible data. The reaction outcome is reported to depend highly on conditions such as protein concentration, size, interactions, surfaces, and other aspects of the reaction setup as well as the complexity of the sample.^53,100^ With the aim of making PICUP a more reproducible and versatile method, we designed a reaction chamber in which the geometry and lighting time can be precisely controlled, and we studied and optimized some of the more important variables affecting the cross-linking reaction.

Our reaction chamber proves to be a valuable tool for performing PICUP. Not only does it allow for a well-controlled and reproducible reaction but it is also easy to produce from affordable materials, with a total cost below 100 $. This makes it an easily accessible tool for other research groups to perform the reaction. At the same time, the reaction chamber has allowed us to reach a millisecond precision in lighting time, decreasing the likelihood of species being cross-linked due to diffusion into random close contacts. This has proven to be crucial for PICUP, and 1 ms lighting was enough to show cross-linked products. Finally, being based in computer-aided design and 3D printing, it is easy to alter the 3D model to fit different sample sizes or tube shapes while maintaining the rest intact.

### 3.2 End of the Reaction and Stopping Reagent

Our findings regarding the termination of the PICUP reaction and the possible stopping reagents also help making the method more easily useable. The fact that the time waited between turning off the light and adding the stop solution did not affect the outcome shows that the reaction is over quite quickly after the light is switched off. This together with the observation of stronger oligomeric bands with longer lighting time implies that the reagents are continuously being activated by light but reactive for only a short time. This means that among the parameters investigated when using PICUP, tight control of the lighting time is the most crucial parameter. On top of that, we have proved that the use of a reducing agent is not strictly necessary (Figure S6). If the PICUP products are analyzed using SDS-PAGE, adding regular loading dye is enough to stop the reaction and preventing it from further happening. These results open new possibilities for the choice of the reaction stopping reagents, based on the compatibility with other methods of choice for analysis or application of cross-linked oligomers.

### 3.3 Importance of Lighting Time

**Figure 9:**
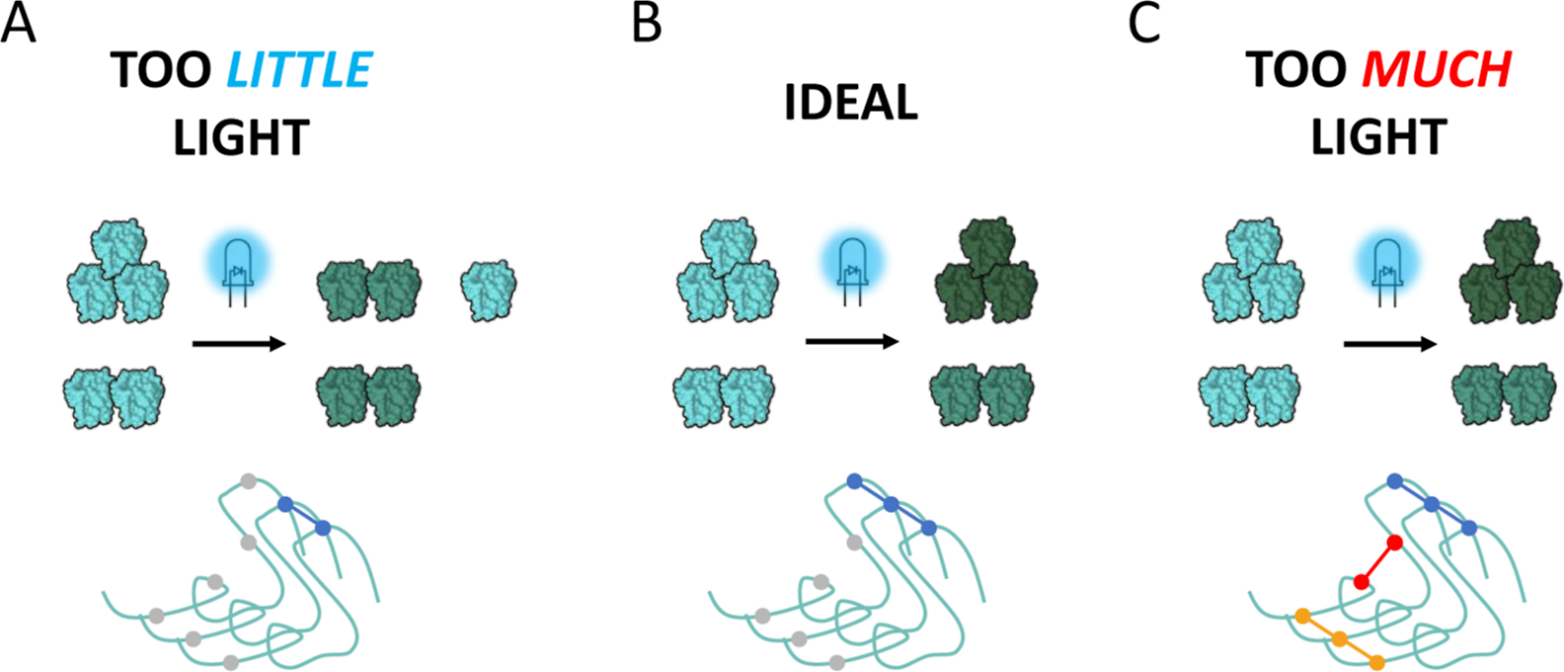
Effect of cross-linking time on the product. In the ideal case (B), every monomer within an oligomer will be cross-linked together, leading to covalently bond oligomers of the same size as those present before the reaction. At the same time, the ideal case would be for a single residue to be cross-linking between monomers, increasing the simplicity of the system, and allowing a better control of it. When the reaction time is too short (A), all monomers within an oligomer might not be cross-linked together, which will bias the system to lower order covalently bond oligomers. Shorter reaction times also mean that it is likelier that only the most reactive residue is cross-linked, but it also risks some otherwise favored cross-links not happening. If the reaction time used is too long (C), the likelihood of cross-linking every monomer within an oligomer is higher, but the risks of cross-linking species that diffuse into temporary close contact increase. Longer reaction time also means that more residues will be activated, creating a more heterogeneous cross-linking pattern as well as internal cross-linking.

The study of a new molecular system would ideally start with testing a range of lighting times to find the most suitable lighting time depending on the purpose of the investigation, which in most cases will be an optimum between advantages and shortcomings of each option (Figure 9). In an ideal case (Figure 9B), every monomer within a non-covalently assembled transient oligomer would become covalently bonded to another monomer, and all monomers in that oligomer would be cross-linked into a single covalent unit (branched or unbranched). If the sample was exposed to denaturing conditions, as is the case for SDS-PAGE, the product would keep the size distribution the sample had before performing the PICUP. In this case, it would also be possible to use PICUP to “freeze” a sample in transient size distribution and to use fractionation methods to enrich each component in the distribution for further study.

However, the higher the number of monomers in an oligomer, the more cross-links between those monomers have to occur for the species to be fully cross-linked. This means that if the employed lighting time is very short (Figure 9A), only some monomers in an assembly may be cross-linked, and most of them are cross-linked to only one other monomer. Therefore, the lower order species are going to be more likely to be fully cross-linked together than the higher order ones, and monomers within higher order assemblies may end up as dimers on the gel. This leads to the system being biased toward lower species in shorter lighting times. On the other hand, longer reaction times (Figure 9C) increase the likelihood of freely diffusing species being cross-linked, potentially forming oligomeric species that are not representative of the sample. On top of that, we have observed that a single millisecond is enough to generate cross-linked dimers. During a long reaction time, initially formed cross-links might affect the continued reaction. Thus, the longer the lighting time, the more species not representative of the system may be formed, and the bigger the deviation from the initial state of interest.

If the cross-linked product is homogeneous in terms of cross-linked residues, this would simplify the further analysis of the sample and allow PICUP to be more easily used for structural interpretations of cross-linked products. The shorter the lighting time applied for PICUP, the higher the odds that only the most reactive residue will be cross-linked. Long lighting times (Figure 9C) increase the likelihood of less reactive residues cross-linking together, and internal cross-linking becomes more likely, increasing the complexity of the sample. This can be observed as a blurriness of the bands, which increases with lighting time (Figure 3).

The potential bias of both the high and the low lighting time seem to indicate that PICUP is not an ideal method for quantification of an oligomer size distribution or the concentration of particular oligomers. However, it is still a quite powerful tool for comparative studies under different conditions or, as in the current study, to compare samples withdrawn from an ongoing reaction at different time points. We conclude that to use the method in a comparative manner, it will be most informative to use a combination of a short and a long lighting time in parallel (i.e., 50 ms and 1 s). This way, if the oligomer pattern can be seen to increase or decrease in both conditions, the comparison can be carried out with more certainty.

### 3.4 Effect of Thioflavin T

Given that many α-synuclein aggregation studies are performed in the presence of ThT, it is crucial to assess whether the presence of the fluorophore alters the oligomeric state of the protein or the PICUP reaction per second. Our results show that at a ThT concentration of 20 μM, which is commonly used in α-synuclein aggregation studies,^94,95^ and sometimes even higher,^101,102^ the population of cross-linked oligomers obtained has clearly increased relative to the reaction without ThT. We cannot, based on our data, discern whether ThT affects the cross-linking reaction itself, or whether ThT favors oligomer formation potentially by binding to certain species and stabilizing them, thus altering the species distribution of the system. Given that the excitation wavelength of 450 nm that is used in PICUP will also excite ThT, the former option may not be excluded, and given the ability of ThT to bind to fibrils,^103^ the latter option may not be disregarded.

### 3.5 α-Synuclein PICUP Products are Oligomers

The optimization of the method allowed us to study α-synuclein and its PICUP products in detail. The fact that we can observe oligomers when performing PICUP on sample freshly purified with SEC makes it necessary to study whether cross-linking is happening between diffusing species. There is some discrepancy in the literature, and some studies find PICUP to cross-link diffusing species,^20,43,47^ while others do not.^38,44^ The variation may reflect the conditions used in the experimental set-up, pH, protein purity, and concentration. This is supported by the fact that Fancy et al.^20^ observe significant cross-linking of 20 μM Lysozyme with 1 s lighting time, whereas we do not. In this study, we used human lysozyme of similar size, that contains more reactive residues compared to α-synuclein, as a control protein under the same experimental conditions as for α-synuclein. However, no cross-linking was observed for lysozyme even if the PICUP reaction was performed with 4 s lighting time (Figure 4), implying that the cross-linked species detected for α-synuclein are not a result of diffusion and random collisions. This supports the idea that the cross-linked products we observe from α-synuclein come from oligomeric species that exist in the solution before performing the PICUP reaction.

Regarding the oligomer distribution, dimers show the highest concentration, with an overall trend of lower concentration the bigger the oligomeric species, although the trend is not perfectly monotonic indicating a higher instability of some oligomeric sizes, as previously reported for Aβ40.^47^ This is the case regardless of the lighting time applied to the reaction (Figure 3), or at which time point in the aggregation process the sample is collected (Figure 7C,D). The same trend was observed in Monte–Carlo simulations of model peptides,^104^ in which the dimer was found to be the most populated species, and the oligomer concentration was lower the larger its size. However, as previously noted, bigger oligomers require more cross-links to remain intact in the SDS PAGE analysis. PICUP’s bias toward smaller species should always be kept in mind when comparing oligomers of different sizes.

### 3.6 α-Synuclein PICUP Oligomers Originate from Monomers

The presence of oligomers directly after SEC implies that these species are formed from monomers. This is supported by the fact that aggregation of α-synuclein clearly leads to a decrease over time and eventually disappearance of these oligomeric species. The emergence of fibrils monitored with ThT fluorescence follows a sigmoidal curve, which is a mirror image of the disappearance of monomers, as reviewed by Arosio et al.^105^ The ImageJ analysis of the bands thus shows that the PICUP cross-linked oligomer concentration follows the time-dependence of the monomer concentration (Figure 7A). This, together with the fact that we observe them directly after SEC purification, suggests that the oligomers we observe are formed from monomers. Their disappearance also follows the appearance of fibrils, which makes it possible that their disappearance is catalyzed by the fibrils. This same behavior was observed in two additional independent measurements (Figure S7).

Interestingly, the only difference observed throughout the aggregation is a decrease in intensity of the bands, and at no point a change in band mobility/height, except the double band below dimer size that appear independently of PICUP. Considering the reported heterogeneity of α-synuclein oligomers, one may expect there to be the same order oligomers (dimers, trimers, etc.) with different cross-linked residues and patterns. If this was the case, these species could have different morphology and thus mobility in SDS-PAGE. The fact that we only observe one clear band at each size position implies either that only one type of oligomer of each size forms or that our methods (PICUP and SDS-PAGE) are only susceptible to one specific structure of oligomeric α-synuclein. Considering that the reaction leads to covalent bond formation between two residues including one with an induced radical, one would expect these residues to be at a covalent bond distance during the lifetime of the induced radical to be able to be cross-linked. The longest observed C–C covalent bond distance to date is 1.806 Å.^106^ This means that the distance requirement is quite restrictive to what residues in an assembly can be cross-linked with each other. Due to that, one could expect a small change in the structure to alter the reaction outcome or even prevent the reaction from occurring. Given this, we believe the most likely scenario to be that PICUP is cross-linking oligomers of a specific structure under our conditions. Thus, our observations apply to PICUP-visible oligomers but not to PICUP-invisible ones.

When separation by sedimentation (at relatively low g-force) was used to separate the solution and fibril phase after PICUP (Figure 8), most oligomers were identified as following the solution phase in the beginning of the aggregation (time point 0). Interestingly, at the end of the reaction, the only oligomeric band visible at 1 s lighting time is a dimer band (∼30 kDa), which clearly follows the fibril phase. The origin of this dimeric band may relate to the process of secondary nucleation. This species could originate from (i) a dimer bound to the fibril surface, or (ii) a peripheral monomer bound to the fibril surface cross-linking with a fibrillar α-synuclein monomer unit. Because its concentration is lowest when the fibril concentration is highest, it less likely originates from (iii) α-synuclein monomers within the fibril structure cross-linking together.

### 3.7 Concluding Remarks

Our reaction chamber design and optimized PICUP shows that lighting time is an important factor to control for reproducible results. The potential bias of different lighting times makes the method best suited for comparative studies. We further conclude that using a short and long lighting time in parallel makes PICUP most informative as a comparative method. By selecting the shortest lighting time where cross-linking is observed, and the highest where many species can be seen without blurriness, one can reach safer conclusions when comparing samples prepared under different conditions. Finally, by comparing samples withdrawn as a function of time of an ongoing aggregation reaction, we find that the α-synuclein oligomers obtained with PICUP are formed from monomeric species: the oligomer concentration disappears in parallel with the monomer concentration and mirrors the appearance of fibrils. The optimization of the PICUP protocol for α-synuclein as presented here may contribute to making the method a valuable tool for studying the complexity and relevance of α-synuclein oligomers in Parkinson’s disease.

## 4 Methods

### 4.1 Protein Expression

Wild-type human α-synuclein was expressed in *Escherichia coli* BL21 DE3 pLysS *_ from a pET-3a-plasmid with an ATG start codon and *E. coli*-optimized codons (purchased from GenScript, Piscataway; NJ). The transformation of the plasmid into *E. coli* was performed by mixing the plasmid with Ca^2+^-competent cells and keeping on ice for 30–60 min. The sample was incubated at 42 °C for 45 s and placed on ice for additional 10 min. Next, the sample was spread onto LB agar plates containing chloramphenicol (30 μg/mL) and ampicillin (50 μg/mL) and incubated at 37 °C overnight (ON). Single small colonies were picked and inoculated in 50 mL of LB media with chloramphenicol (30 μg/mL) and ampicillin (50 μg/mL) ON with shaking. The morning after, 5 mL of each cell culture was transferred into pre-heated (37 °C) 500 mL of LB medium in a 2.5 L baffled flask with continues shaking at 125 rpm. The cell culture was induced with 100 μg/mL isopropyl β-d-1-thiogalactopyranoside when the optical density at 600 nm had reached 0.9–1.0. 4 hours later, the cells were harvested by using centrifugation at 6000*g* at 4 °C (JA 8.100 rotor) for 12 min. Cells obtained from 4 L culture were combined and mixed with 25 mL of water and stored at −20 °C until purification. Before harvesting, 1 mL of samples was taken from the cultures to test for the efficiency of the expression (procedure described in more detail by Pálmadóttir et al.).^107^

### 4.2 Purification of α-Synuclein

The cell pellet obtained from 8 L of cell culture was thawed in 100–120 mL of cold buffer A [10 mM Tris/HCl, 1 mM ethylenediaminetetraacetic acid, pH 7.5]. The pellet was sonicated into a homogeneous sample, using pulse sonication (1 s on, 1 s off) with the beaker inserted in an ice–water slurry to keep the sample cold during sonication. After sonication, the sample was centrifuged for 10 min at 15,000*g* at 4 °C (JA 25.50 rotor). The supernatant was collected and poured into an equal volume of boiling buffer A. The sample was continuously stirred until the temperature had reached 85 °C, then placed on ice with stirring until the sample had cooled. The sample was centrifuged again to pellet the precipitated *E. coli* proteins. The supernatant was collected and used for further purification by ion-exchange chromatography. A column with a diameter of 3.5 cm containing 100 g of diethylaminoethyl (DEAE) cellulose was equilibrated in buffer A. The supernatant was loaded onto the column, which was then washed with 100 mL of buffer A. Next, the sample was eluted with a linear salt gradient of 0–0.5 M NaCl gradient in buffer A and total gradient volume 1400 mL at a flow rate of 1 mL/min. SDS-PAGE was used to analyze which fractions contained α-synuclein. Fractions containing α-synuclein were pooled and purified using a second ion-exchange column consisting of 60 g of wet DEAE sephacel resin in a column with diameter of 2.3 cm, performed in the same way as before. The absorbance at 280 nm of the different fractions was measured. The fractions showing absorbance at 280 nm were further analyzed with SDS-PAGE. The fractions containing the peak for α-synuclein and no contaminants were combined and stored as 1 mL of aliquots at −20 °C. The concentration of the pooled sample (in the range between 1 and 3 mg/mL) was determined by absorbance spectroscopy using a NanoDrop instrument (average over 9 repeats). The purity was further investigated using MALDI-TOF mass spectrometry (see the Supporting Information) (the purification procedure is described in more detail by Pálmadóttir et al.).^107^

### 4.3 Monomer Isolation

Size exclusion chromatography was performed to isolate the α-synuclein monomers prior to experiments. Aliquots obtained after the second ion-exchange step were lyophilized and then dissolved in 1 mL of 6 M guanidinium hydrochloride. The samples were incubated for 1 h at room temperature and separated on a Superdex 75 Increase 10/300 GL (GE Healthcare) column using a fast liquid protein chromatography system (Bio-RAD, BioLogic Duo Flow, USA). The sample was eluted in the running buffer [10 mM MES/NaOH, 0.02% (w/v) NaN_3_, pH = 5.5] at 0.7 mL/min. The elution of the monomeric peak was followed by absorbance at 280 nm, and the center of the monomeric peak (∼1 mL) was collected in low binding tubes (Genuine Axygen Quality). The concentration of the pure sample was determined by absorbance at 280 nm, using an extinction coefficient of ε_280_ = 5800 M^–1^ cm^–1^. The quality of the sample was further investigated by MALDI-TOF mass spectrometry (see the Supporting Information) and by performing aggregation kinetic analysis of the batch. Samples used for kinetic experiments were freshly prepared and kept on ice until the start of each experiment (approximately 10 min). Samples used for PICUP experiments regarding the effect of lighting time, reaction termination, and the effect of ThT concentration were kept on ice for approximately 0.5–1 h before the PICUP reaction due to the duration of preparation steps.

### 4.4 Aggregation Kinetics

The aggregation kinetics of α-synuclein were monitored by following thioflavin T (ThT) fluorescence in a 96-well half area low-binding PEG-coated polystyrene plate with transparent bottom (3881 Corning). Seed fibrils were used to trigger the aggregation process. The seeds were formed by incubating α-synuclein in a low-binding tube with a magnetic stir bar at 37 °C. After 2 days, samples were collected, aliquoted, and frozen. Before usage, seed aliquots were taken from the freezer, thawed, and placed in a sonication bath for 1 min before incubation at RT for 1 h.

Aggregation kinetics were followed by incubating a mixture with a final concentration of 20 μM monomeric α-synuclein, 3 μM ThT, and 200 nM seeds (generated from 20 μM monomeric α-synuclein and diluted 100 times in the mixture) in MES buffer [10 mM MES, 0.02% (w/v) = 3 mM NaN_3_, pH 5.5]. The monomeric α-synuclein was prepared directly before the experiment, as indicated before (see Section 3.3). 100 μL of the mixture was loaded in each well in a 96-well half area low-binding PEG-coated polystyrene plate with a transparent bottom (3881 Corning). The plate was sealed with a transparent SealPlate film to avoid evaporation and incubated at 37 °C without shaking in a FLUOstar Omega plate reader (BMG Labtech, Offenburg, Germany). The aggregation was followed by measuring the ThT fluorescence at different time points, with excitation and emission wavelengths of 448 and 480 nm, respectively, and 100 nm band pass filter in each case. The fluorescence was measured for approximately 20–25 h, or until the aggregation curve reached a plateau. Samples were collected at different time points from the 96-well plate for immediate execution of the PICUP reaction.

### 4.5 Separation of Fibrils and Species in Solution

For separation of α-synuclein fibrils and the remaining species (monomers and oligomers), the sample was centrifuged at 18,000 rpm for 2 min with a MIKRO 220R centrifuge. 80 μL of the supernatant was collected and kept as the fraction with the species in solution. The remaining supernatant was discarded. The pellet was resuspended in 100 μL of MES buffer [10 mM MES/NaOH, 0.02% (w/v) NaN_3_, pH = 5.5] and centrifuged again at 18,000 rpm for 2 min. After getting rid of the supernatant, the pellet was resuspended in 100 μL of MES buffer and kept as the pellet fraction containing fibrils.

### 4.6 Building the Reaction Chamber

The design of the instrument was based on the most common conditions used in preciously published studies using PICUP. These include the use of standard polymerase chain reaction (PCR) tubes (Multiply-Pro 0.2 mL Biosphere) as the reaction container and a total reaction volume of 20 μL.^15,20,23,26,31,34,38−40,42−44,47−49,54−56,58,59,62−67,73,76^ In addition, the casing was also designed to fit a LED450-06 LED from Roithner LaserTechnik GmbH at short distance (1 mm) from the tube wall (Figures 2 and S8).

#### 4.6.1 3D Printing of Casing

The final model was built into a standard triangle language (stl) file using the ZW3D computer-aided design and manufacturing system (CAD/CAM) (stl file can be found here). This file was then imported and sliced with Eiger software and printed with a Markforged Onyx One 3D printer. The material of choice was Onyx carbon reinforced nylon.

#### 4.6.2 Programming

The computer program for the reaction chamber was written in Arduino. The code was written in Arduino language, a set of C/C++ functions. The code was written in a way so the lighting time can be defined by the user prior to each reaction (Arduino code can be found here).

#### 4.6.3 Mounting

The Arduino code was run through a Pololu A-Star 32U4 Mini SV (ac02c) board, where a LED450-06 LED (450 nm wavelength) was connected to the board together with a 470 Ω resistor to ensure a 20 mA current (Figure 2). Finally, the board and its components were mounted with the casing to complete the reaction chamber. The code was written to control the lighting time through the computer via Arduino’s serial monitor. Due to that, the computer and the Arduino board had to be connected with a USB-to-micro-HDMI adaptor.

### 4.7 Photo-Induced Cross-Linking of Unmodified Proteins

#### 4.7.1 Preparation of Reagents

Reagents were dissolved and diluted in 10 mM MES/NaOH buffer at pH 5.5. Approximately 1 mg of Ru(bpy) was dissolved in the correct volume of the MES buffer to obtain 5 mM Ru(bpy) and then further diluted to 1 mM Ru(bpy) in the MES buffer. A similar procedure was carried out for APS, where approximately 10 mg was dissolved in MES buffer to obtain 100 mM APS, and then further diluted to 20 mM APS. Both reagents were aliquoted into 25 μL of fractions and frozen until use.

For most experiments, β-mercaptoethanol was used to stop the PICUP reaction as a part of the 5× STOP buffer. The 5× STOP buffer was prepared by mixing 4 mL of 4.5 M Tris pH 8.45 buffer with 4.8 mL of glycerol and 1 g of SDS. The mixture was dissolved by heating it up in a boiling water bath. When the mixture became transparent and homogeneous, 1 mL of β-mercaptoethanol was added. Finally, 1% coomassie blue was added in varying amounts between 0.2 and 1 mL. The mixture was aliquoted in fractions of 100 μL and frozen. Prior to use, aliquots were thawed by exposure to warm water until a homogeneous liquid was obtained.

#### 4.7.2 Cross-Linking Reaction

18 μL of protein solution (20 μM) was mixed in a PCR tube with 1 μL of Ru(bpy) (1 mM) and 1 μL of APS (20 mM), for a final Ru(bpy)/protein ratio of ∼3:1. After mixing, the tube was placed in the reaction chamber and exposed to light for the desired time, as previously set in the program. The reaction was stopped by addition of the 5× STOP buffer. Before loading the sample onto the gel, the mixture was thoroughly pipetted up and down to ensure proper mixing. Reaction controls were carried out by either adding buffer instead of the reagents or by adding the reagents but not exposing the sample to light. Every experiment was carried out in a dark room with minimal light to reduce errors in light exposure time. The control for PICUP of diffusing species was performed with human lysozyme (L1667, Sigma-Aldrich). The protein was purchased at >90% purity and therefore needed further purification using two steps [cation exchange chromatography in 10 mM Tris, pH = 7.5 buffer on a HiTrap CM Sepharose Fast Flow (Cytiva) column; and size exclusion chromatography in 10 mM MES/NaOH, 0.02% (w/v) NaN_3_, pH = 5.5 buffer on a Superdex 75 Increase 10/300 GL (GE Healthcare) column].

#### 4.7.3 SDS PAGE Analysis

Once the reaction was stopped, samples were evaluated by SDS-PAGE. Novex 10–20% tricine pre-cast gels were used together with their corresponding tricine SDS running buffer [100 mM Tris base, 100 mM tricine, 0.1% (w/v) SDS, pH 8.3]. As the 5× STOP buffer was used for most experiments, mixtures were directly loaded into the gel after stopping the reaction. The PageRuler prestained protein ladder was used to evaluate the sample’s molecular weight. The electrophoresis was performed at 70 mV for 15 min, followed by an increase in voltage up to a maximum of 120 mV until the dye reached the bottom of the gel. Gels were stained either with InstantBlue or with Silver Staining. InstantBlue was chosen over coomassie because, in contrast with other staining solutions, InstantBlue stains the proteins but not the gel, leading to a better signal-to-noise ratio, crucial for comparative intensity analysis of the bands. Protein bands were visible within 15 min with InstantBlue, but overnight staining allowed for full sensitivity. Finally, an optional incubation of the gel in water removed any background coloring. The gels were scanned with an Epson Expression 10000XL scanner. The images were analyzed with ImageJ.^108^

PICUP reaction chamber stl file and Arduino code can be found free of charge at https://github.com/LOrtigosa/PICUP

## Supporting Information Available

PICUP control reaction; PICUP of α-synuclein with different lighting times; example of ImageJ analysis of SDS-PAGE gel bands; photo-induced cross-linking of lysozyme under different lighting times; effect of ThT on the PICUP of lysozyme; effect of β-mercaptoethanol in stopping PICUP; PICUP of α-synuclein at different points throughout its aggregation; model used for the 3D-printed PICUP reaction chamber; and mass spectrometry analysis of Asp119-truncated synuclein (PDF)
