# Supplementary material for: Photo-Induced Cross-Linking of Unmodified α-Synuclein Oligomers
Source: ACS Chem Neurosci. 2023 Aug 25;14(17):3192–205. doi: 10.1021/acschemneuro.3c00326 (PMC10485903; doi:10.1021/acschemneuro.3c00326)
Supplement: Supplementary file 1 — cn3c00326_si_001.pdf [file cn3c00326_si_001.pdf]

Supporting information

Photo-induced crosslinking of unmodified α-synuclein oligomers

Lei Ortigosa-Pascual*, Thom Leiding, Sara Linse, and Tinna Pálmadóttir*

Department of Biochemistry and Structural Biology, Lund University, 221 00 Lund, Sweden
Email: lei.ortigosa@biochemistry.lu.se ; tinna.palmadottir@biochemistry.lu.se

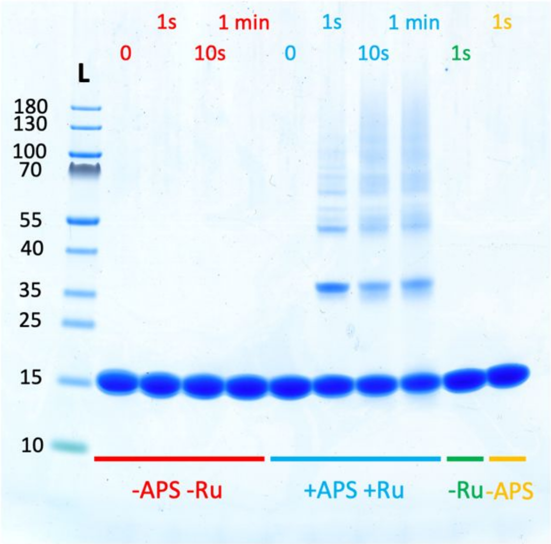

Figure S1. PICUP control reaction. PICUP of α-synuclein was performed missing APS (-APS, yellow), missing Ru(bpy) (-Ru, green), missing both (-APS-Ru, red), or containing both (+APS+Ru, blue). The reaction was done for the lighting time indicated above each corresponding lane.

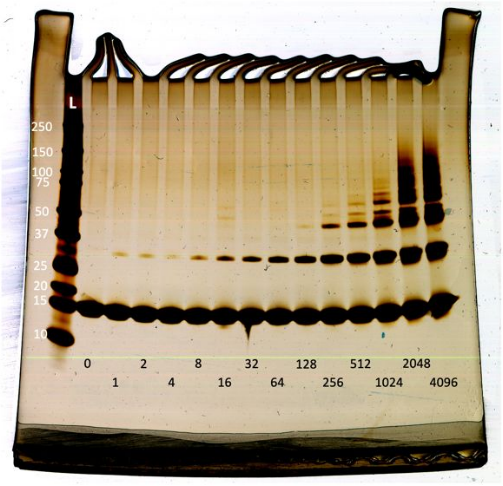

Figure S2. PICUP of α-synuclein with different lighting times, indicated below lanes (ms). The gel is the same as showed in Figure 3 of the main body of the article, but the saturation of the Epson Expression 10000XL scanner was turned to the highest possible while allowing to see the bands. Even at this extreme, no species other than that of monomer size (~15 kDa) were visible when light was not applied (0).

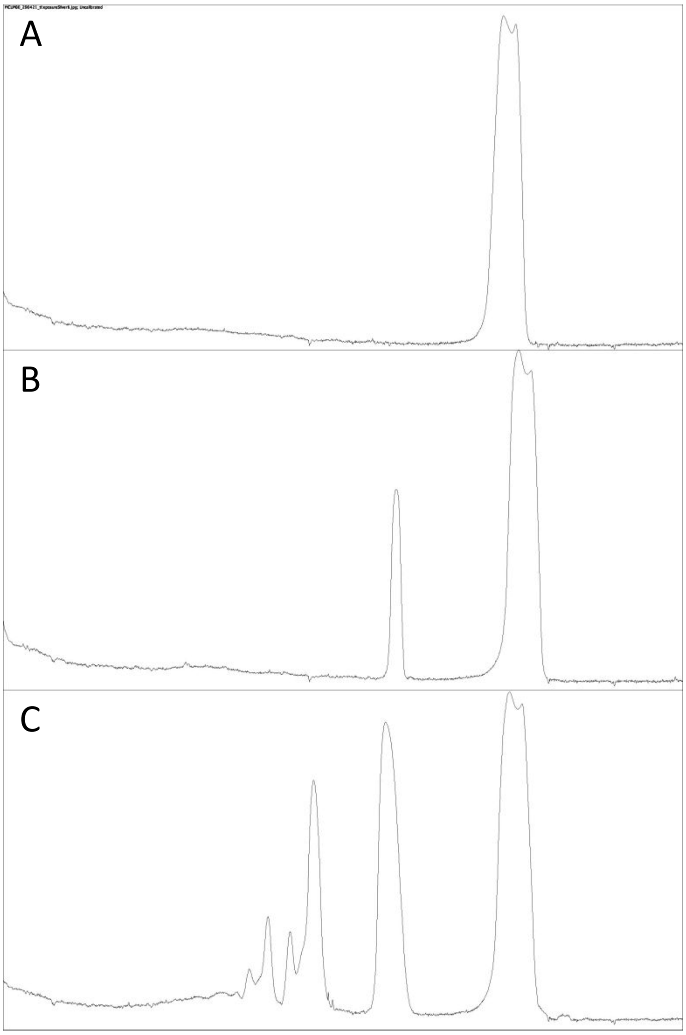

Figure S3. Example of ImageJ analysis of SDS-PAGE gel bands. α-synuclein was cross-linked at different lighting times and the sample was run in an SDS-PAGE gel and stained with Silver Staining (Figure 3B). In ImageJ, a previously selected lane of the gel can be plotted as the intensity of the pixels in the figure over the selected area. The examples above are from the lanes with 0 ms (A), 64 ms (B) and 1024 ms (C) lighting time. While the 0 ms reaction only shows one peak representing the monomer band, longer PICUP times lead to the formation of bigger order bands, seen as additional peaks in this representation. After setting a baseline, the area below each peak is measured, and the fraction of oligomers relative to the total amount of species (oligomers plus monomer peak) is calculated with those values.

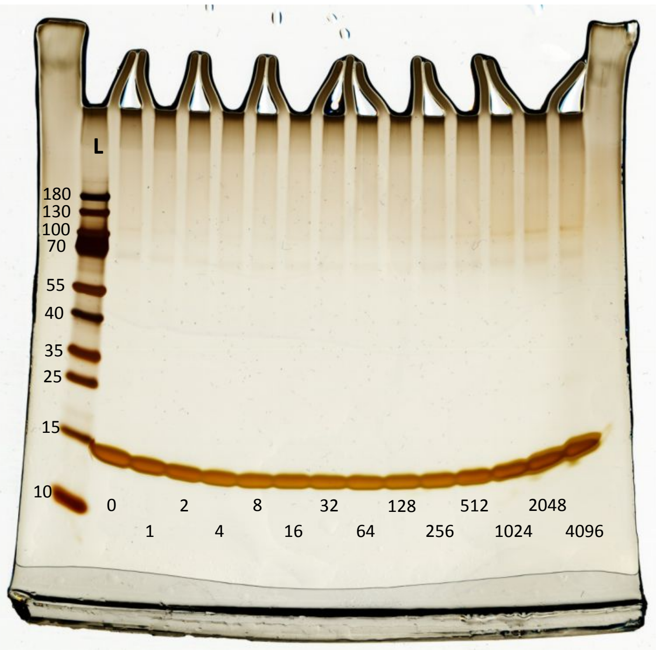

Figure S4. Photo induced crosslinking of lysozyme under different lighting times. The number under each lane indicates the lighting time in ms. The samples run in this gel were the same as those run in the gel at Figure 4B, but this gel was stained with silver stain for higher resolution.

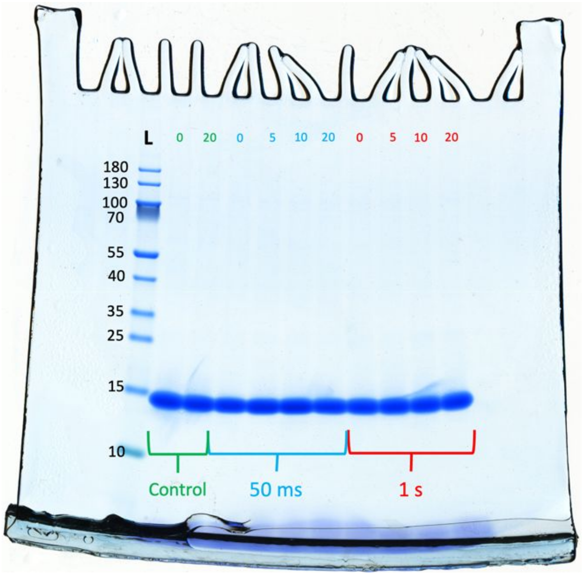

Figure S5. Effect of ThT on the PICUP of Lysozyme. Lysozyme was crosslinked in the presence of different concentrations of ThT (0, 0.3, 1, 3, 9 and 20 μM), indicated at the top of each lane. The reaction was performed with both 50 ms (blue bracket) and 1 s (red bracket) lighting times. Two controls with either 0 or 20 μM ThT were performed where buffer was added instead of the PICUP reagents, and no light was applied (green bracket).

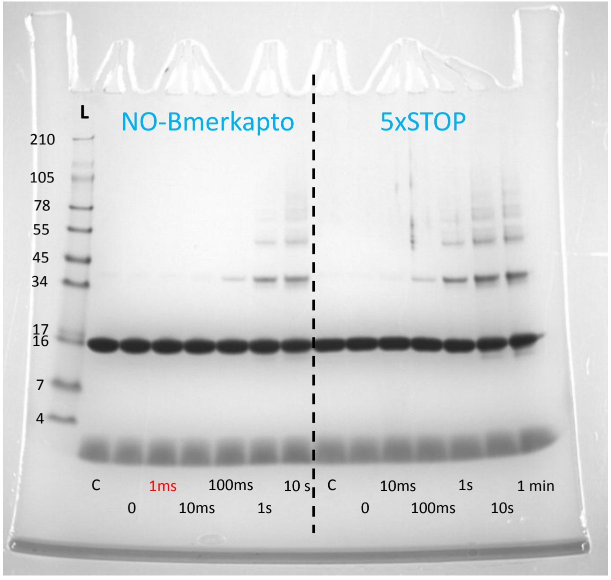

Figure S6. PICUP of α-synuclein stopped with different stopping reagents. The reaction was performed at lighting times between 0 ms and 1 min (indicated below each lane). Controls were done by adding buffer instead of the reagents (C). Finally, the reaction was stopped by either 5xSTOP buffer (5xSTOP) or by a buffer prepared the same way but substituting β-mercaptoethanol with Tris buffer (NO-Bmerkapto). It should be noted that the non-β-mercaptoethanol sample has an additional lighting time (1ms, in red) between the 0 and 10 ms samples which the 5xSTOP sample doesn't have.

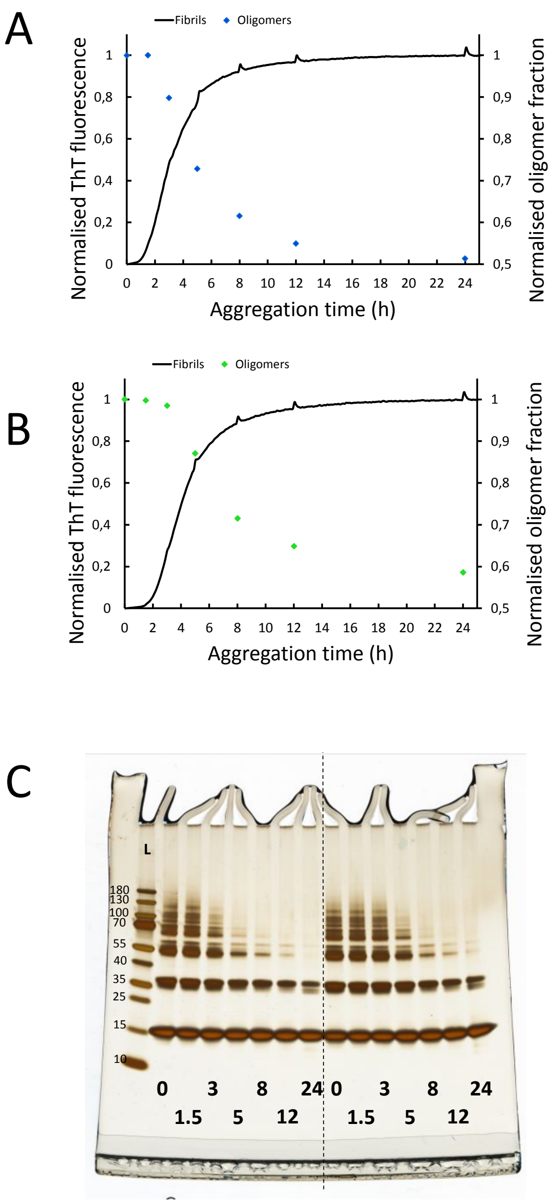

Figure S7. PICUP of α-synuclein at different points throughout its aggregation. Two parallel experiments were run where 20 μM monomeric α-synuclein was mixed with 200 nM seeds from two different sources. Their aggregation was monitored at 37° C by following the fluorescence of 20 μM ThT. The fluorescence of 5 samples of each preparation were normalized (A, light orange dots; B, light pink dots), and their average was plotted as a function of time (black line). Samples were collected at t = 0, 1.5, 3, 5, 8, 12 and 24 h, cross-linked for 1 s, and analyzed with SDS-PAGE stained with silver staining (C, left samples correspond to aggregation in A, right samples to aggregation in B). ImageJ analysis of the bands was performed, and the oligomer fraction was calculated by dividing the intensity of oligomeric bands by the total intensity of all bands in the lane. Oligomer fractions were then normalized relative to the value at t = 0 (A, blue dots; B, green dots) and plotted with the ThT curve.

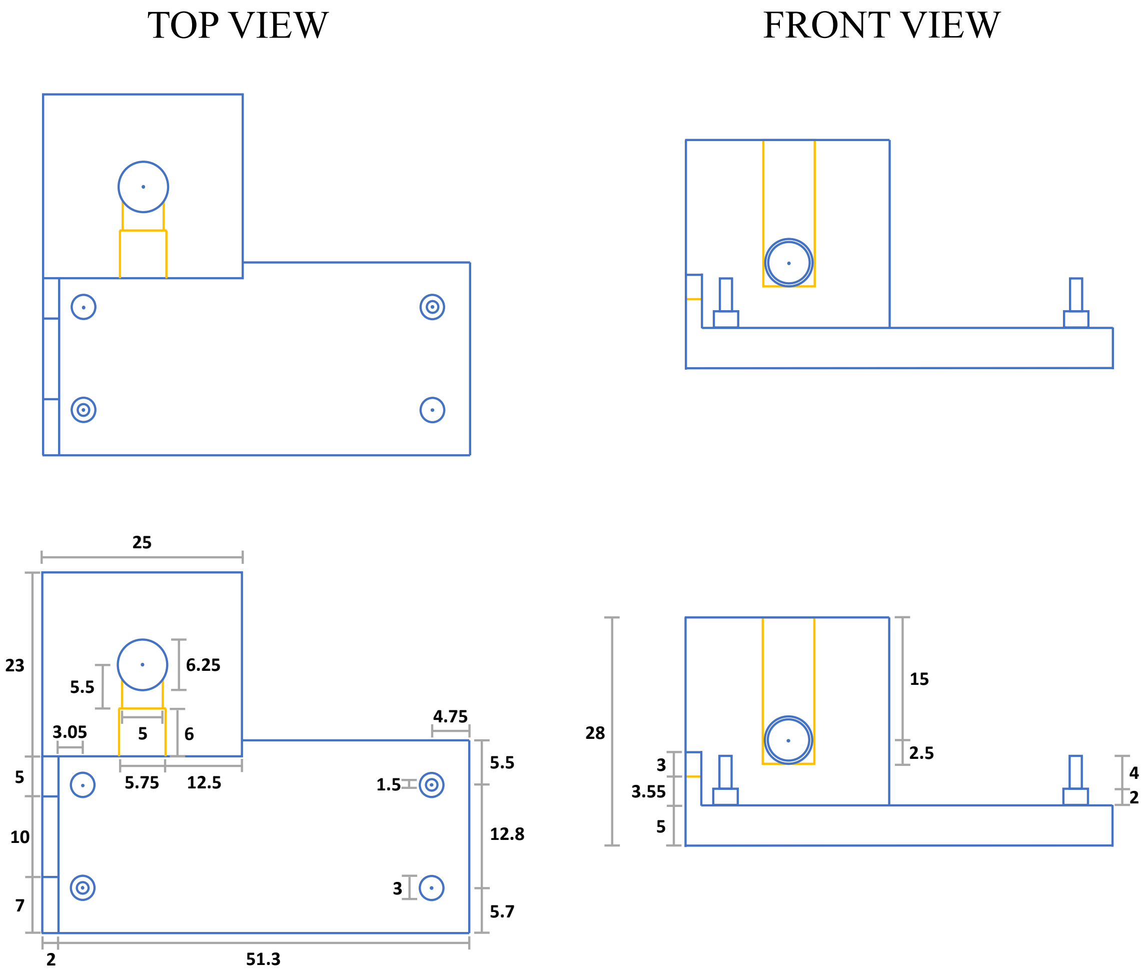

Figure S8. Model used for the 3D printed PICUP reaction chamber with (bottom) and without (top) measurements. Stl file available at https://github.com/LOrtigosa/PICUP.

Mass spectrometry analysis of Asp119 truncated α-synuclein

Sample digested with Trypsin led to the detection of α-synuclein fragment 103-119 (NEEGAPQEGILEDMPVD).

1 MDVFMKGLSK AKEGVVAAAE KTKQGVAEAA GKTKEGVLYV GSKTKEGVVH
51 GVATVAEKTK EQVTNVGGAV VTGVTAVAQK TVEGAGSIAA ATGFVKKDQL
101 GK**NEEGAPQE GILEDMPVD**

Monoisotopic mass of neutral peptide: 1857.7938 Da was detected as a 2+ charged peptide at m/z 929.9033. MS/MS fragmentation of 929.9033 m/z peptide led to the detection of the following b and y ions (in red):

b ion #	m/z	Sequence	m/z	y ion #
b1	115.050	**N**		y17
b2	244.093	**E**	1744.758	y16
b3	373.135	**E**	1615.716	y15
b4	430.157	**G**	1486.673	y14
b5	501.194	**A**	1429.652	y13
b6	598.247	**P**	1358.614	y12
b7	726.305	**Q**	1261.562	y11
b8	855.348	**E**	1133.503	y10
b9	912.369	**G**	1004.460	y9
b10	1025.453	**I**	947.439	y8
b11	1138.537	**L**	834.355	y7
b12	1267.580	**E**	721.271	y6
b13	1382.607	**D**	592.228	y5
b14	1529.642	**M_{ox}**	477.201	y4
b15	1626.695	**P**	330.166	y3
b16	1725.764	**V**	233.113	y2
b17		**D**	134.045	y1

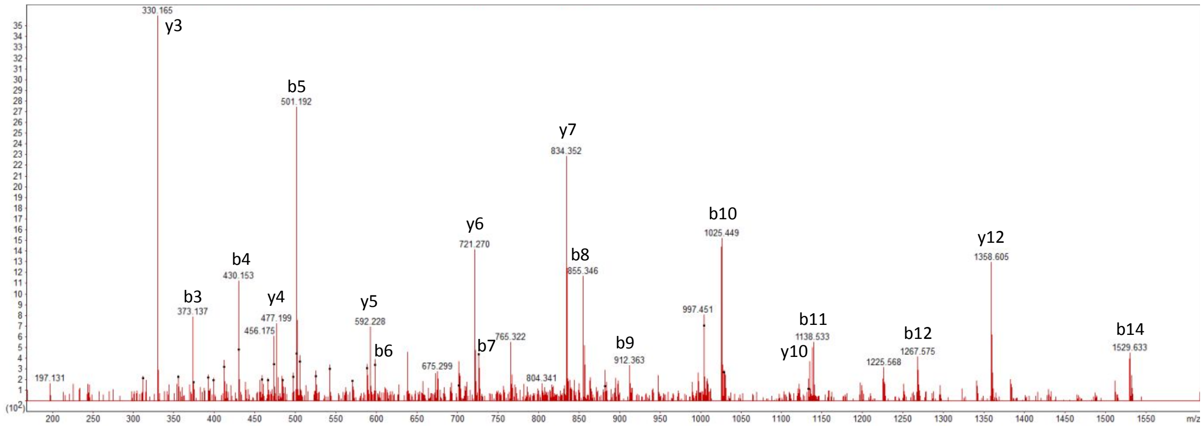

MS/MS spectra of 929.9033 m/z peptide, leading to the detection of b and y ions indicated on table above.
